# Effects of Fas-mediated liver cell apoptosis on diethylnitrosamine-induced hepatocarcinogenesis in mice

**DOI:** 10.1054/bjoc.1999.0944

**Published:** 2000-01-18

**Authors:** A Hara, N Yoshimi, Y Yamada, K Matsunaga, K Kawabata, S Sugie, H Mori

**Affiliations:** Department of Pathology, Gifu University School of Medicine, 40 Tsukasamachi, Gifu, 500-8705, Japan

**Keywords:** Fas, apoptosis, diethylnitrosamine, mouse, hepatocarcinogenesis

## Abstract

The present study was designed to investigate the effect of Fas-mediated liver cell apoptosis, induced by a hamster monoclonal antibody against mouse Fas antigen, on diethylnitrosamine (DEN)-induced hepatocarcinogenesis in mice. DEN (10 μg g^–1^, intraperitoneally (i.p.)) was given to 15-day-old male C3H/HeJ mice. Three weeks after DEN treatment, Fas-mediated liver cell apoptosis induced by anti-Fas antibody resulted in a biphasic effect on induction of liver cell tumours, depending on dosage and time of antibody administration. Single or multiple treatment with high dose anti-Fas antibody (5 μg animal^−1^), induced gross liver cell damage and decreased the incidence of liver cell tumours in DEN-treated mice. In contrast, five treatments with low dose anti-Fas antibody (2 μg animal^−1^), induced dispersed localized liver cell damage and promoted the number of large-sized liver cell adenomas and hepatocellular carcinomas. These findings suggest that high dose anti-Fas antibody has a marked effect on the clearance of DEN-initiated liver cells, whereas repeated administration of low dose anti-Fas antibody promotes hepatocarcinogenesis. It is concluded that Fas-mediated liver cell apoptosis has a biphasic effect on hepatocarcinogenesis. © 2000 Cancer Research Campaign


*British Journal of Cancer* (2000) **82**(2), 467–471
© 2000 Cancer Research Campaign
Article no. bjoc.1999.0944

### Effects of Fas-mediated liver cell apoptosis on diethylnitrosamine-induced hepatocarcinogenesis in mice

**A Hara, N Yoshimi, Y Yamada, K Matsunaga, K Kawabata, S Sugie and H Mori**

Department of Pathology, Gifu University School of Medicine, 40 Tsukasamachi, Gifu 500-8705, Japan

**Summary** The present study was designed to investigate the effect of Fas-mediated liver cell apoptosis, induced by a hamster monoclonal antibody against mouse Fas antigen, on diethylnitrosamine (DEN)-induced hepatocarcinogenesis in mice. DEN (10 μg $g^{-1}$, intraperitoneally (i.p.)) was given to 15-day-old male C3H/HeJ mice. Three weeks after DEN treatment, Fas-mediated liver cell apoptosis induced by anti-Fas antibody resulted in a biphasic effect on induction of liver cell tumours, depending on dosage and time of antibody administration. Single or multiple treatment with high dose anti-Fas antibody (5 μg $animal^{-1}$), induced gross liver cell damage and decreased the incidence of liver cell tumours in DEN-treated mice. In contrast, five treatments with low dose anti-Fas antibody (2 μg $animal^{-1}$), induced dispersed localized liver cell damage and promoted the number of large-sized liver cell adenomas and hepatocellular carcinomas. These findings suggest that high dose anti-Fas antibody has a marked effect on the clearance of DEN-initiated liver cells, whereas repeated administration of low dose anti-Fas antibody promotes hepatocarcinogenesis. It is concluded that Fas-mediated liver cell apoptosis has a biphasic effect on hepatocarcinogenesis. © 2000 Cancer Research Campaign

**Keywords**: Fas; apoptosis; diethylnitrosamine; mouse; hepatocarcinogenesis

Viral infection such as hepatitis C or B virus is the major cause for chronic hepatitis or liver cirrhosis, which enhances the risk of developing hepatocellular carcinomas (Dusheiko, 1990; Matsuda et al, 1995). It has been demonstrated that apoptotic cell death of liver cells occurs in acute or chronic hepatitis and that Fas-mediated liver cell apoptosis accounts for T-cell-mediated cytotoxicity against liver cells (Hiramatsu et al, 1994; Mita et al, 1994). Up-regulation of Fas occurs in acute or chronic liver diseases due to viral infections (Okazaki et al, 1996; Ando et al, 1997; Saile et al, 1997). It has been suggested that apoptotic cell death induced by Fas/Fas ligand system plays a critical role in the clearance of virus-infected liver cells (Mochizuki et al, 1996; Hayashi and Mita, 1997). Thus, apoptotic cell death of virus-infected hepatocytes is an integral component of the disease process in chronic hepatitis or liver cirrhosis (Jiang et al, 1997).

In animal models, sustained or repeated liver cell damage induced by frequent partial hepatectomy or carbon tetrachloride administration is known to stimulate cell proliferation and influence the promotion phase of hepatocarcinogenesis, thereby increasing the number of preneoplastic lesions and tumours (Pound and McGuire, 1978*a*; Lindroos et al, 1991). It has been hypothesized that Fas-mediated apoptosis, induced by Fas ligand expressed by cytotoxic T-cell in chronic hepatitis or liver cirrhosis, is critical step in hepatocyte proliferation and promotion of hepatocarcinogenesis. Recently, it has been reported that intraperitoneal administration of monoclonal anti-Fas antibody (which possesses cytolytic activity against cells expressing mouse Fas antigen) induces apoptotic liver cell death (Ogasawara et al, 1993; Nishimura et al, 1997).

In the present study, we examined the in vivo effects of Fas-mediated apoptosis, induced by intraperitoneal (i.p.) administration of a monoclonal antibody against mouse Fas antigen, on DEN-induced hepatocarcinogenesis of C3H/HeJ mouse hepatocytes. We also evaluated the effect of this antibody on acute liver cell damage, using the terminal dUTP-biotin nick end labelling (TUNEL) method (Gavrieli et al, 1992).

*Received 25 February 1999*
*Revised 7 July 1999*
*Accepted 9 July 1999*

*Correspondence to:* A Hara

### MATERIALS AND METHODS

### Mice

C3H/HeJ mice were purchased from Japan SLC Inc. (Hamamatsu, Japan). Only male C3H/HeJ mice were used in this study. The animals were housed in plastic cages under controlled conditions of 23 ± 2°C, 50 ± 10% humidity and a 12 h light/dark cycle. They were fed commercial pellet CE-2 diet (Nippon Clea Co., Tokyo, Japan). The animal studies were carried out with the approval of our institutional committee of the care and use of laboratory animals.

### Evaluation of acute liver damage induced by anti-Fas antibody

The acute liver damage 12 h after i.p. administration of Jo2 antibody at high (5 μg $animal^{-1}$) or low (2 μg $animal^{-1}$) dose was assessed histologically using haematoxylin and eosin (H & E) staining and TUNEL methods. The rationale for using these doses was based on preliminary studies showing that administration of

Jo2 at 10 and 100 μg animal$^{-1}$ in C3H/HeJ mice resulted in 50% and 0% survival respectively, and that 5 and 2 μg animal$^{-1}$ Jo2 antibody treatment induced gross or scattered liver cell damage respectively. Male C3H/HeJ mice at 5 weeks of age received a single i.p. administration of Jo2 antibody at a dose of 5 or 2 μg animal$^{-1}$ in physiological saline. The mice were sacrificed 12 h after the treatment, and the livers were removed and prepared for a histological examination. Five animals were used for each dose of the antibody to analyse liver damage. Five animals were also used as negative controls.

### In situ labelling of DNA fragmentation of acute liver damage

The TUNEL assay was performed using some modification of the method of Gavrieli et al (Gavrieli et al, 1992; Hara et al, 1995*a*; 1995*b*). After incubation with 20 μg ml$^{-1}$ proteinase K (Sigma), the liver sections were immersed in TDT buffer (30 mM Trizma base, pH 7.2, 140 mM sodium cacodylate, 1 mM cobalt chloride). TDT (Boehringer Mannheim GmbH, Mannheim, Germany) and biotinylated dUTP (Boehringer Mannheim GmbH) were diluted in TDT buffer at concentrations of 0.15 e.u. μl$^{-1}$ and 0.8 nmol respectively. The solution was placed on the sections, and then incubated at 37°C for 60 min. The sections were covered with streptavidin peroxidase (Dako Co., Carpinteria, CA, USA), and stained with 3,3′-diaminobenzidine as a substrate for the peroxidase. Finally, counter staining was done using Mayer's haematoxylin. The percentage of TUNEL-positive cells in liver was determined by measuring the number of liver cells possessing dark-brown nuclei, as a proportion of the entire cell number of liver parenchymal cells.

### Fas-mediated apoptosis on DEN-induced hepatocarcinogenesis

At 14 days of age, a total of 125 male C3H/HeJ mice (group 1–5) received i.p. injection of DEN (Sigma Chemical Co., St Louis, MO, USA) dissolved in physiological saline at a dose of 10 μg g$^{-1}$ body weight. All mice were weaned 3 weeks after birth. Control mice (group 6–8) at the same age received i.p. injection of physiological saline. At 5 weeks of age, groups 1, 2 and 6 were treated i.p. with hamster monoclonal antibody (Jo2 antibody) against mouse Fas antigen (Pharmingen, San Diego, CA, USA) at a dose of 5 μg animal$^{-1}$ in physiological saline. Group 1 ($n$ = 24) received a single administration of this antibody, whereas groups 2 ($n$ = 22) and 6 ($n$ = 20) received five i.p. injections at every 3 weeks. Groups 3, 4 and 7 were treated i.p. with Jo2 antibody (2 μg animal$^{-1}$ in physiological saline). Group 3 ($n$ = 24) received single administration of the antibody, whereas groups 4 ($n$ = 25) and 7 ($n$ = 21) received five i.p. injections every 3 weeks. Group 5 ($n$ = 25) received only i.p. injection of DEN, and group 8 ($n$ = 10) was non-treated control. The survival rate of mice receiving one dose of 5 μg animal$^{-1}$ of Jo2 antibody was 90%, while 100% of animals survived the 2 μg animal$^{-1}$ dose. The mice that did not survive the antibody treatment died within 12 h after the injection. Histologically, acidophilic cytoplasm, pyknotic cell nuclei and severe haemorrhage were present in the livers of all the mice that died from injection of 5 μg animal$^{-1}$ of Jo2 antibody. In Groups 2 and 6, no mice died following four injections of 5 μg animal$^{-1}$ of Jo2 antibody.

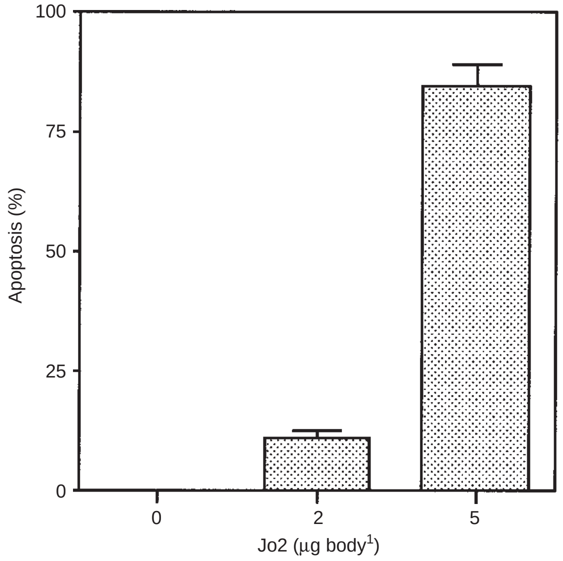

**Figure 1** Mean apoptotic rates of Jo2-induced acute liver damage 12 h after i.p. administration of Jo2 antibody at the doses of 0, 2 and 5 μg animal$^{-1}$. Apoptotic rate was determined by measuring the number of TUNEL-positive liver cells as a proportion of the entire cell number of liver parenchymal cells

### Studies of liver tumours

At 40 weeks of age, all mice were killed by ether anaesthesia. Livers were excised, weighed and examined for the presence of visible lesions. The number and location of the lesions were recorded. Livers were sliced at 2-mm intervals, following fixation in 10% phosphate-buffered formalin for 48 h. The number of grossly visible lesions were reconfirmed in the sliced livers. The formalin-fixed livers were dehydrated and embedded in paraffin for histological studies.

### Histological analysis

From each paraffin-embedded specimen, 3-μm-thick sections were cut. Deparaffinized sections were stained with H&E. The incidences of hepatocellular carcinomas and adenomas were histologically discriminated and quantified by two pathologists using criteria defined previously (Frith and Ward, 1979; Lipsky et al, 1981; Klaunig et al, 1987). Furthermore, adenomas were classified into two subgroups; small-sized adenomas which are less than 5 mm in diameter and large-sized adenomas that are more than 5 mm in diameter. Differences of liver tumour incidences among the groups were analysed statistically by Student's $t$-test.

### RESULTS

### Acute liver damage induced by Jo2

Histologically, livers of mice 12 h after exposure to 5 μg animal$^{-1}$ Jo2 antibody (high dose), showed many areas of focal haemorrhage, and abundant hepatocytes with eosinophilic swollen cytoplasm and pyknotic nuclei. Only hepatocytes localized in the centrolobular regions survived. In TUNEL staining, positive reaction was evident in most hepatocytes associated with apoptotic cell nuclei, although a few non-apoptotic cells remained in the centrolobular regions. In contrast, acidophilic cells with pyknotic

*© 2000 Cancer Research Campaign*

**Table 1** Tumour induction in DEN-initiated mice treated with Jo2 anti-Fas antibody

| Group | DEN | Dose of Jo2 (μg animal$^{-1}$) | No of times of Jo2 treatment | No. of mice | Mean no. of liver tumours per mouse | Mean no. of adenomas (< 5 mm) per mouse | Mean no. of adenomas (> 5 mm) per mouse | Mean no. of carcinomas per mouse |
|---|---|---|---|---|---|---|---|---|
| 1 | + | 5 | 1 | 24 | 6.0 ± 5.4[a,b] | 5.0 ± 3.7[c] | 1.0 ± 2.3 | 0.1 ± 0.3 |
| 2 | + | 5 | 5 | 22 | 5.6 ± 4.5[c] | 4.6 ± 2.6[c] | 1.0 ± 2.3 | 0.0 ± 0.0 |
| 3 | + | 2 | 1 | 24 | 10.2 ± 7.9 | 7.4 ± 5.9 | 2.8 ± 3.3 | 0.1 ± 0.2 |
| 4 | + | 2 | 5 | 25 | 14.6 ± 11.0 | 9.7 ± 5.2 | 4.0 ± 5.5[d] | 0.9 ± 1.5[e] |
| 5 | + | – | – | 25 | 10.9 ± 5.0 | 9.2 ± 3.8 | 1.6 ± 2.4 | 0.1 ± 0.3 |
| 6 | – | 5 | 5 | 20 | 0.0 ± 0.0 | 0.0 ± 0.0 | 0.0 ± 0.0 | 0.0 ± 0.0 |
| 7 | – | 2 | 5 | 21 | 0.0 ± 0.0 | 0.0 ± 0.0 | 0.0 ± 0.0 | 0.0 ± 0.0 |
| 8 | – | – | – | 10 | 0.0 ± 0.0 | 0.0 ± 0.0 | 0.0 ± 0.0 | 0.0 ± 0.0 |

[a] Values are mean ± s.d. [b,c,d,e] Significantly different from mice treated with DEN alone by Student's *t*-test ([b]$P < 0.01$, [c]$P < 0.001$, [d]$P < 0.05$, [e]$P < 0.02$).

nuclei were found scattered throughout the liver of mice treated with 2 μg animal$^{-1}$ Jo2 antibody (low dose). In addition, no specific zonal distribution of these cells was noted. TUNEL staining revealed that apoptotic cells were distributed sparsely in the liver tissue. Control mice without Jo2 treatment had neither damaged hepatocytes nor apoptotic cells in the liver. The mean number of apoptotic cells per 500 hepatocytes of mice at the high dose and low dose of Jo2, and that of negative controls were 84.4 ± 9.3, 10.6 ± 3.8 and 0 ± 0 respectively (Figure 1).

### Development of liver tumours

As summarized in Table 1, the liver tumours were only detected in the groups treated with DEN (groups 1–5). All the neoplasms were histologically determined as having a hepatocellular origin. Jo2 antibody administered five times at high (group 6) or low (group 7) dose, without DEN administration, induced no liver tumours.

### Effects of Jo2 at high dose on DEN-induced hepatocarcinogenesis

Total number of liver tumours of mice receiving single (group 1) or multiple (group 2) treatments of high dose Jo2 antibody was significantly lower ($P < 0.01$ and $P < 0.001$ respectively) than that of mice treated with DEN alone (group 5). Importantly, the number of adenomas < 5 mm in diameter in both groups 1 and 2 were less than that of group 5 ($P < 0.001$), indicating that high dose Jo2 antibody markedly reduced the DEN-induced liver tumours, irrespective of the frequency of the administration and that the suppressive effect was apparent in small-sized adenomas.

### Effects of Jo2 at low dose on DEN-induced hepatocarcinogenesis

In contrast to high dose Jo2, the effect of low dose antibody given 5 times (group 4) significantly increased both the number of adenomas > 5 mm in diameter ($P < 0.05$), and also carcinomas ($P < 0.02$). Treatment of Jo2 had no effect on the number of adenomas < 5 mm in diameter (group 5). The single administration of Jo2 (group 3), however, showed no effect on the number of small- and large-sized adenomas and carcinomas. These findings suggest that frequent administration of low dose of Jo2 increased the DEN-induced liver tumours and that the effect of Jo2 was apparent in large-sized adenomas and carcinomas.

### DISCUSSION

Fas, which is a member of the tumour necrosis factor/nerve growth factor receptor family (Smith et al, 1994), is expressed in varied types of tissues, particularly in liver (Watanabe-Fukunaga et al, 1992; Leithauser et al, 1993). Fas-mediated apoptosis may serve to eliminate liver cells in which hepatocarcinogenesis has been initiated by carcinogens. However, carcinogen-exposed liver cells which survive Fas-mediated programmed cell death may acquire a potential for neoplastic transformation due to a regenerative response of the remaining liver tissue. Hence, it could be argued that differences in sensitivity of initiated and non-initiated liver cells to Fas-ligand results in the cells developing different potentials for neoplastic transformation. In fact, the differential sensitivity to Fas-mediated apoptosis has been demonstrated for both carcinoma cells and normal liver cells (Higaki et al, 1996).

In this study, dose-related liver cell damage by Jo2 antibody in male C3H/HeJ mice was evaluated. High dose of Jo2 antibody generated hepatocytes with typical characteristics of apoptosis, namely eosinophilic swollen cytoplasm, pyknotic nuclei and apoptotic bodies. These morphological features appeared to be coincident with those of the human fulminant hepatitis (Ogasawara et al, 1993). In contrast, low dose of Jo2 antibody produced acidophilic cells with pyknotic nuclei throughout the liver tissue without specific zonal distribution. Such finding is similar to that of human chronic hepatitis (Jiang et al, 1997). The apoptotic liver cell death was confirmed by TUNEL staining.

Interestingly, high dose of Jo2 antibody suppressed DEN-induced liver tumours independent of the frequency (single or 5 times) of administration. The suppressive effect of Jo2 occurred primarily in small-sized adenomas, and while the numbers of large-sized adenomas and carcinomas were also decreased by this treatment the effects did not reach statistical significance. Thus, while gross liver damage by Fas-mediated apoptosis reduced the number of liver tumours, the process of the hepatocarcinogenesis itself may not be markedly affected. On the other hand, low dose of Jo2 antibody increased the number of large-sized adenomas and carcinomas, and the effect was particularly apparent with multiple administration of the antibody. This suggests that frequent or chronic induction of sparsely-distributed apoptotic cell death in liver provokes the development of large-sized adenomas and carcinomas, or the malignant progression of liver neoplasms.

Difference of the sensitivity to induction of Fas-mediated apoptosis between normal hepatocytes and neoplastic cells or virus-infected cells has previously been reported. It is known that

*© 2000 Cancer Research Campaign*

some human neoplastic liver cells show resistance to Fas-mediated apoptosis in vitro (Natoli et al, 1995). SV40 T-antigen contributing to viral pathogenesis is also known to protect the infected liver cells against the host apoptotic defence mechanism (Rouquet et al, 1995). Furthermore, Higaki et al (1996) demonstrated a relationship between Fas expression and apoptotic cell numbers in human hepatocellular carcinomas and related liver tissues. They established that apoptotic cell counts were significantly higher in Fas-expressing tissues than in Fas-negative tissues, and that hepatocellular carcinoma tissues expressed Fas less frequently and more weakly than noncancerous tissues. These findings imply that neoplastic liver cells may be less sensitive to Fas-mediated apoptosis than non-neoplastic liver cells. In the present study, high dose of Jo2 antibody suppressed occurrence of liver tumours, whereas low dose promoted the tumorigenesis. Our data suggest that high dose of Jo2 antibody induces apoptosis in both non-initiated and initiated liver cells, but low dose of Jo2 antibody produces apoptosis in only non-initiated liver cells, which makes initiated cells survive and regenerate after the frequent exposure to low dose Jo2 antibody.

Tumour-suppressing effects are recognized not only in Fas-mediated apoptosis but apoptosis induced by chemical agents. It is hypothesized that lead nitrate decreases the number of hyperplastic lesions in DEN-treated rats by inducing apoptotic cell death of initiated hepatocytes (Columbano et al, 1991). Contrary to apoptosis-related suppressing effects, it is known that stimulation of cell proliferation greatly influences the promotion phase of carcinogenesis, and then increases the number of preneoplastic lesions and tumours (Pound and McGuire, 1978*a*, 1978*b*; Dragani et al, 1986; Lindroos et al, 1991). Liver is essentially a quiescent organ with respect to hepatocyte proliferation. Liver cell proliferation is usually stimulated by partial hepatectomy or carbon tetrachloride-induced acute liver damage in rodent models of hepatocarcinogenesis (Pound and McGuire, 1978*a*; Lindroos et al, 1991). Regenerative growth following liver injury has been shown to promote hepatocarcinogenesis in mice (Pound and McGuire, 1978*b*; Dragani et al, 1986). Our results suggests that low dose Jo2 antibody induces the regenerative growth following apoptotic liver cell injury and then promotes DEN-induced hepatocarcinogenesis.

In conclusion, our results suggest that high dose of anti-Fas antibody induces gross liver cell apoptosis and plays an important role in the clearance of DEN-initiated liver cells, and that frequent treatment of low dose of anti-Fas antibody generates scattered liver cell apoptosis and exert a promoting effect on the DEN-induced hepatocarcinogenesis. Accordingly, Fas-mediated liver cell apoptosis plays a biphasic effect on hepatocarcinogenesis. Further studies are warranted to elucidate the difference of sensitivity of initiated and non-initiated liver cells to Fas/Fas ligand system.

### ACKNOWLEDGEMENTS

The authors wish to thank Mr Kazumasa Sato for animal care, and Ms Kyoko Takahashi, Ms Chikako Usui and Ms Tomoko Kajita for technical assistance.

### REFERENCES

Ando K, Hiroishi K, Kaneko T, Moriyama T, Muto Y, Kayagaki N, Yagita H, Okumura K and Imawari M (1997) Perforin, Fas/Fas ligand, and TNF-alpha pathways as specific and bystander killing mechanisms of hepatitis C virus-specific human CTL. *J Immunol* **158**: 5283–5291

Columbano A, Endoh T, Denda A, Noguchi O, Nakae D, Hasegawa K, Ledda-Columbano GM, Zedda AI and Konishi Y (1996) Effects of cell proliferation and cell death (apoptosis and necrosis) on the early stages of rat hepatocarcinogenesis. *Carcinogenesis* **17**: 395–400

Dragani TA, Manenti G and Della Porta G (1986) Enhancing effects of carbon tetrachloride in mouse hepatocarcinogenesis. *Cancer Lett* **31**: 171–179

Dusheiko GM (1990) Hepatocellular carcinoma associated with chronic viral hepatitis. Aetiology, diagnosis and treatment. *Br Med Bull* **46**: 492–511

Frith CH and Ward JM (1979) A morphologic classification of proliferative and neoplastic hepatic lesions in mice. *J Environmen Pathol Toxicol* **3**: 329–351

Gavrieli Y, Sherman Y and Ben-Sasson SA (1992) Identification of programmed cell death in situ via specific labeling of nuclear DNA fragmentation. *J Cell Biol* **119**: 493–501

Hara A, Yoshimi N, Hirose Y, Ino N, Tanaka T and Mori H (1995*a*) DNA fragmentation in granular cells of human cerebellum following global ischemia. *Brain Res* **697**: 247–250

Hara A, Yoshimi N, Mori H, Iwai T, Sakai N, Yamada H and Niwa M (1995*b*) Hypothermic prevention of nuclear DNA fragmentation in gerbil hippocampus following transient forebrain ischemia. *Neurol Res* **17**: 461–464

Hayashi N and Mita E (1997) Fas system and apoptosis in viral hepatitis. *J Gastroenterol Hepatol* **12**: S223–226

Higaki K, Yano H and Kojiro M (1996) Fas antigen expression and its relationship with apoptosis in human hepatocellular carcinoma and noncancerous tissues. *Am J Pathol* **149**: 429–437

Hiramatsu N, Hayashi N, Katayama K, Mochizuki K, Kawanishi Y, Kasahara A, Fusamoto H and Kamada T (1994) Immunohistochemical detection of Fas antigen in liver tissue of patients with chronic hepatitis C. *Hepatology* **19**: 1354–1359

Jiang Z, Liu Y, Savas L, Smith L, Bonkovsky H, Baker S and Banner B (1997) Frequency and distribution of DNA fragmentation as a marker of cell death in chronic liver diseases. *Virchows Arch* **431**: 189–194

Klaunig JE, Weghorst CM and Pereira MA (1987) Effect of the age of B6C3F1 mice on phenobarbital promotion of diethylnitrosamine-initiated liver tumors. *Toxicol Appl Pharmacol* **90**: 79–85

Leithauser F, Dhein J, Mechtersheimer G, Koretz K, Bruderlein S, Henne C, Schmidt A, Debatin KM, Krammer PH and Moller P (1993) Constitutive and induced expression of APO-1, a new member of the nerve growth factor/tumor necrosis factor receptor superfamily, in normal and neoplastic cells. *Lab Invest* **69**: 415–429

Lindroos PM, Zarnegar R and Michalopoulos GK (1991) Hepatocyte growth factor (hepatopoietin A) rapidly increases in plasma before DNA synthesis and liver regeneration stimulated by partial hepatectomy and carbon tetrachloride administration. *Hepatology* **13**: 743–750

Lipsky MM, Hinton DE, Klaunig JE and Trump BF (1981) Biology of hepatocellular neoplasia in the mouse. III. Electron microscopy of safrole-induced hepatocellular adenomas and hepatocellular carcinomas. *J Natl Cancer Inst* **67**: 393–405

Matsuda Y, Amuro Y, Higashino K, Hada T, Yamamoto T, Fujikura M, Yamaguchi K, Shimomura S, Iijima H and Nakano T (1995) Relation between markers for viral hepatitis and clinical features of Japanese patients with hepatocellular carcinoma: possible role of alcohol in promoting carcinogenesis. *Hepato-Gastroenterol* **42**: 151–154

Mita E, Hayashi N, Iio S, Takehara T, Hijioka T, Kasahara A, Fusamoto H and Kamada T (1994) Role of Fas ligand in apoptosis induced by hepatitis C virus infection. *Biochem Biophys Res Com* mun **204**: 468–474

Mochizuki K, Hayashi N, Hiramatsu N, Katayama K, Kawanishi Y, Kasahara A, Fusamoto H and Kamada T (1996) Fas antigen expression in liver tissues of patients with chronic hepatitis B. *J Hepatol* **24**: 1–7

Natoli G, Ianni A, Costanzo A, De Petrillo G, Ilari I, Chirillo P, Balsano C and Levrero M (1995) Resistance to Fas-mediated apoptosis in human hepatoma cells. *Oncogene* **11**: 1157–1164

Nishimura Y, Hirabayashi Y, Matsuzaki Y, Musette P, Ishii A, Nakauchi H, Inoue T and Yonehara S (1997) In vivo analysis of Fas antigen-mediated apoptosis: effects of agonistic anti-mouse Fas mAb on thymus, spleen and liver. *Int Immunol* **9**: 307–316

Ogasawara J, Watanabe-Fukunaga R, Adachi M, Matsuzawa A, Kasugai T, Kitamura Y, Itoh N, Suda T and Nagata S (1993) Lethal effect of the anti-Fas antibody in mice. *Nature* **364**: 806–809

Okazaki M, Hino K, Fujii K, Kobayashi N and Okita K (1996) Hepatic Fas antigen expression before and after interferon therapy in patients with chronic hepatitis C. *Digest Dis Sci* **41**: 2453–2458

Pound AW and McGuire LJ (1978*a*) Repeated partial hepatectomy as a promoting stimulus for carcinogenic response of liver to nitrosamines in rats. *Br J Cancer* **37**: 585–594

© 2000 Cancer Research Campaign

Pound AW and McGuire LJ (1978*b*) Influence of repeated liver regeneration on hepatic carcinogenesis by diethylnitrosamine in mice. *Br J Cancer* **37**: 595–602
Rouquet N, Allemand I, Molina T, Bennoun M, Briand P and Joulin V (1995) Fas-dependent apoptosis is impaired by SV40 T-antigen in transgenic liver. *Oncogene* **11**: 1061–1067
Saile B, Knittel T, Matthes N, Schott P and Ramadori G (1997) CD95/CD95L-mediated apoptosis of the hepatic stellate cell. A mechanism terminating uncontrolled hepatic stellate cell proliferation during hepatic tissue repair. *Am J Pathol* **151**: 1265–1272
Smith CA, Farrah T and Goodwin RG (1994) The TNF receptor superfamily of cellular and viral proteins: activation, costimulation, and death. *Cell* **76**: 959–962
Watanabe-Fukunaga R, Brannan CI, Itoh N, Yonehara S, Copeland NG, Jenkins NA and Nagata S (1992) The cDNA structure, expression, and chromosomal assignment of the mouse Fas antigen. *J Immunol* **148**: 1274–1279

*© 2000 Cancer Research Campaign*
